# Introducing COCOS: codon consequence scanner for annotating reading frame changes induced by stop-lost and frame shift variants

**DOI:** 10.1093/bioinformatics/btw820

**Published:** 2017-01-18

**Authors:** Mariusz Butkiewicz, Jonathan L Haines, William S Bush

**Affiliations:** Department of Epidemiology and Biostatistics, Institute for Computational Biology, Case Western Reserve University, Cleveland, OH, USA

## Abstract

**Summary:**

Reading frame altering genomic variants can impact gene expression levels and the structure of protein products, thus potentially inducing disease phenotypes. Current annotation approaches report the impact of such variants in the context of altered DNA sequence only; attributes of the resulting transcript, reading frame and translated protein product are not reported. To remedy this shortcoming, we present a new genetic annotation approach termed Codon Consequence Scanner (COCOS). Implemented as an Ensembl variant effect predictor (VEP) plugin, COCOS captures amino acid sequence alterations stemming from variants that produce an altered reading frame, such as stop-lost variants and small insertions and deletions (InDels). To highlight its significance, COCOS was applied to data from the 1000 Genomes Project. Transcripts affected by stop-lost variants introduce a median of 15 amino acids, while InDels have a more extensive impact with a median of 66 amino acids being incorporated. Captured sequence alterations are written out in FASTA format and can be further analyzed for impact on the underlying protein structure.

**Availability and Implementation:**

COCOS is available to all users on github: https://github.com/butkiem/COCOS

## 1 Introduction

Annotation of the functional consequences of DNA sequence variants is increasing in importance for human genomic studies. Specifically, variants that are thought to be highly disruptive to protein sequence (often called Loss-of-Function variants) and are presumed to be a primary driver of disease pathogenesis have garnered special attention. The designation ‘Loss-of-Function’ varies between studies, but generally involves the insertion of a stop-codon, disruption of a splice site and insertions/deletions (InDels) that disrupt the translational reading frame (MacArthur *et al.*, 2012). Many of the resulting transcripts may undergo nonsense-mediated decay (NMD) (Nagy and Maquat, 1998), but recent studies suggest that the majority of these transcripts escape NMD (Lappalainen *et al.*, 2013), and thus their true impact on protein function and human phenotypes remains unknown. Current approaches to annotation (such as those implemented in SNPEff (Cingolani *et al.*, 2012) and Ensembl Variant Effect Predictor (VEP) (McLaren *et al.*, 2010)) report the impact of a variant in the context of altered DNA sequence only; attributes of the resulting transcript, reading frame and altered translated protein product are not reported. Extensions and plugins have been developed to report additional information, such as the LOFTEE plugin for VEP, (https://github.com/konradjk/loftee) which reports/filters stop-gained and frameshift variants only within the last 5% of the transcript and variants within non-canonical splice sites. To alleviate these limitations, we introduce COCOS, ***Co****don****Co****nsequence****S****canner*, a VEP plugin to provide additional information about the altered reading frame and translated sequence resulting from stop-lost variants and small InDels.

## 2 Approach

The primary functionality of the COCOS plugin is to capture AA sequence alterations stemming from variants that produce an altered reading frame, such as stop-lost variants and InDels. Though their potential to dramatically change the ultimate protein sequence is high, some of these variants may be benign, introducing only a few altered AAs in the final coding sequence. The coding sequence is terminated when a subsequent stop-codon terminates the translation process. The COCOS algorithm is outlined in Table 1. Implemented as a plugin of the variant effect predictor (VEP) framework, this design comes with several features. The plugin takes advantage of the continuous updates of VEP and associated databases or caches. Also, resulting captured sequences are provided in FASTA format. Information about the number of altered amino acids (AAs) as well as the length of the original transcript (in percent) are printed directly to the VEP output file as part of the ‘Extra’ field.

**Table 1:** COCOS algorithm

Determine position of variant within transcript. Ignore transcripts where variant does not fall into an exon, variant affects splicing, or variant is located within the 3′ untranslated region (UTR). Given the variant is completely localized within a single exon, concatenate all exon sequences of the reference spliced transcript, including the 3′ UTR. Generate an alternate version of the spliced transcript that contains the variant. Pairwise comparison of each codon in reference and alternate transcript and report any codons that are different UNTIL a Stop Codon is reached on the alternate transcript. Translate reported codons into Amino Acids (AAs). If a Stop Codon is never reached, report this transcript as probably non-viable.

## 3 Application example – COCOS analysis of stop-lost variants and short InDels

**Fig. 1:**
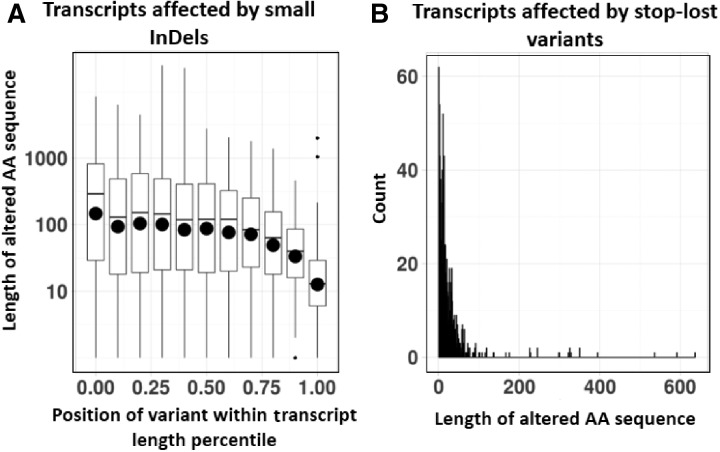
A) shows the distributions of the length of altered AAs with respect to their position in the transcripts affected by small InDels on a logarithmic scale. The horizontal bars denote average values and the larger black dots represent localized median values. B) shows a histogram of the length of AA sequence extensions as an elongation to transcripts affected by stop-lost variants

We accessed data from the 1000 Genomes project (phase 3) (1000 Genomes Project Consortium and others, 2015). A subset of 1283 stop-lost single nucleotide variants and 21 234 exonic short InDels within this dataset were extracted. Of these, 892 stop-lost variants/2115 transcripts (4803 InDels/10 981 transcripts) were identified with viable translation sequence alterations. Table 1 describes the COCOS algorithm and the underlying selection criteria to identify potentially *viable translation sequence alterations*. Figure 1A highlights the location of InDels on transcripts affected and the resulting AA change. The overall median (average) length of changed AAs is 66 (277). Stop-lost variants introduce an extension to the translated AA sequence (Fig. 1B). A median (average) of 15 (26) AAs is introduced as an elongation to the translated transcript sequence.

## 4 Discussion

The COCOS plugin for VEP captures variant induced codon alterations and presents this altered sequence as translated AAs, allowing for improved interpretation or scoring of predicted variant consequences in a protein product disruption context. As seen in Figure 1, these changes can have a substantial effect on transcripts, if translated. Effects induced by stop-lost variants tend to be more benign, due to elongating the translated transcript, compared to effects from small InDels that predominantly take place within the transcript. Variants that alter start codons (start-lost) or create a stop codon (stop-gained) introduce a truncation rather a sequence substitution or extension, and thus are not within the scope of this work. One possible extension is the application of methods that infer biological function of the altered sequence. Assessment for newly introduced protein domains can be of interest or determination of functional peptides like small signaling proteins. Determining the extent of transcript alterations can improve *in silico* annotations of deleterious variants, and thus help interpret implications in a biological context.
